# Differences in the occurrence and abundance of batoids across an oceanic archipelago using complementary data sources: Implications for conservation

**DOI:** 10.1002/ece3.8290

**Published:** 2021-11-18

**Authors:** Fernando Tuya, Ricardo Aguilar, Fernando Espino, Nestor E. Bosch, Eva K. M. Meyers, David Jiménez‐Alvarado, Jose J. Castro, Francisco Otero‐Ferrer, Ricardo Haroun

**Affiliations:** ^1^ Grupo en Biodiversidad y Conservación IU‐ECOAQUA Universidad de Las Palmas de Gran Canaria Las Palmas Spain; ^2^ Oceana Madrid Spain; ^3^ School of Biological Sciences Oceans Institute University of Western Australia Crawley Western Australia Australia; ^4^ Zoologisches Forschungsmuseum Alexander Koenig Bonn Germany

**Keywords:** Atlantic Ocean, chondrichthyes, elasmobranchs, island biogeography, macroecology, rays

## Abstract

Batoids, distributed from shallow to abyssal depths, are considerably vulnerable to anthropogenic threats. Data deficiencies on the distribution patterns of batoids, however, challenge their effective management and conservation. In this study, we took advantage of the particular geological and geomorphological configuration of the Canary Islands, across an east‐to‐west gradient in the eastern Atlantic Ocean, to assess whether patterns in the occurrence and abundance of batoids varied between groups of islands (western, central, and eastern). Data were collected from shallow (<40 m, via underwater visual counts and by a local community science program) and deep waters (60–700 m, via ROV deployments). Eleven species of batoids, assessed by the IUCN Red List of Threatened Species, were registered, including three “Critically Endangered” (*Aetomylaeus bovinus*, *Dipturus batis*, and *Myliobatis aquila*), three “Endangered” (*Gymnura altavela*, *Mobula mobular*, and *Rostroraja alba*), two “Vulnerable” (*Dasyatis pastinaca* and *Raja maderenseis*), and two “Data Deficient” (*Taeniurops grabata* and *Torpedo marmorata*). Also, a “Least Concern” species (*Bathytoshia lata*) was observed. Overall, batoids were ~1 to 2 orders of magnitude more abundant in the central and eastern islands, relative to the western islands. This pattern was consistent among the three sources of data and for both shallow and deep waters. This study, therefore, shows differences in the abundance of batoids across an oceanic archipelago, likely related to varying insular shelf area, availability of habitats, and proximity to the nearby continental (African) mass. Large variation in population abundances among islands suggests that “whole” archipelago management strategies are unlikely to provide adequate conservation. Instead, management plans should be adjusted individually per island and complemented with focused research to fill data gaps on the spatial use and movements of these iconic species.

## Graphical abstract

Differences in the abundance of batoids across an oceanic archipelago suggest that “whole” archipelago management strategies are unlikely to provide adequate conservation. Management plans should be adjusted individually per island.

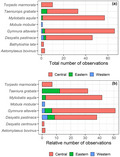

## 1 INTRODUCTION

Batoids, including electric rays, stingrays, shovelnose rays, and skates, are a group of flat‐bodied carnivorous and detritivorous fishes, globally distributed from shallow waters to abyssal depths. Batoids are the largest subgroup of the Chondrichthyes class (around 680 accepted species, Weigmann, 2016), most of which are threatened due to their life‐history traits (e.g., slow growth and low fecundity) coupled with increasing anthropogenic threats, such as overfishing, bycatch, and habitat degradation (Baum & Myers, 2004; Dulvy et al., 2014; Follesa et al., 2019; Martins et al., 2018; Stevens et al., 2000). In fact, five of the seven most threatened Chondrichthyan families worldwide belong to the Batoidea superorder (Dulvy et al., 2014). In 2018, the International Union for Conservation of Nature (IUCN) assessed a total of 573 batoid species, of which ~21% are threatened with extinction (www.iucnredlist.org). A substantial number (~41.7%) of batoids is, moreover, “Data Deficient” (www.iucnredlist.org), which means that their distribution patterns and habitat uses, among other key ecological information, are largely unknown (Dulvy et al., 2014; Flowers et al., 2016, 2021). More data on this fish fauna are, therefore, essential to implement management actions and select priority areas for conservation (Dulvy et al., 2014; Edgar et al., 2020; Le Port et al., 2012; Martins et al., 2018).

Oceanic islands are places of unique biodiversity, which can even connect distant marine populations by creating ecological corridors across oceans (Hobbs et al., 2010; Joyeux et al., 2001; Mazzei et al., 2021). Archipelagos often encompass islands with varying geological and geomorphological histories, which may affect the type and availability of nearshore habitats and, consequently, the abundance and diversity of marine fauna, including elasmobranchs (Das & Afonso, 2017; Mejía‐Falla et al., 2020). Distances and depths among adjacent islands, which affect their isolation, can affect successive colonization events by marine biota, particularly for species of limited pelagic dispersal (Hachich et al., 2020).

The Canary Islands have long been considered a hotspot for batoids and certain nearshore shark species. A recent species checklist identified 24 batoid species in this region (Báez et al., 2019; Appendix S1). For example, the archipelago is considered a unique stronghold of the iconic angelshark, *Squatina squatina* (Linnaeus, 1758) (Barker et al., 2016; Lawson et al., 2019; Meyers et al., 2017). Large aggregations of elasmobranchs are spotted near the shore, including urban beaches (Escánez et al., 2016; Jiménez‐Alvarado et al., 2020; Tuya et al., 2020). Importantly, sea‐cage fish farms act as aggregating structures for a range of rays across times and islands (Dempster et al., 2005; Tuya et al., 2005, 2006). Located west off the African coast, at 28º latitude, this archipelago comprises seven islands and five islets that emerged after successive volcanic events during the last ~20 million years, covering a surface area of 7490 km^2^ and a coastline of 1501 km. The easternmost part of the archipelago (Fuerteventura) is ~95 km away from the shore of the African continent, while La Palma Island is almost ~400 km away (Fernández‐Palacios & Martín‐Esquivel, 2002). Differences in the composition and abundance of marine species across the Canary Island archipelago have been previously observed for both coastal fishes (Tuya et al., 2004) and macroalgae (Tuya & Haroun, 2009). Initially, this was attributed to the large‐scale oceanographic variability associated with the east‐to‐west gradient of the Canary Islands from its closest point to the shore of Africa. While the eastern side of the Canary Islands is influenced by the seasonal coastal upwelling off the African coast, the western part of the archipelago is toward the oligotrophic “open” ocean, under more tropical conditions, and an average SST of 2°C higher (Davenport et al., 2002).

Proximity to the African coast, however, may have concurrently influenced past and present colonization events by epi‐benthic megafauna across the Canarian archipelago; for example, this has been considered as a plausible explanation for the lower frequency of angelshark occurrences toward the westernmost islands (Meyers et al., 2017). In addition, the older islands are located in the eastern and central part of the archipelago (Table 1); these islands have wider insular shelfs compared with the younger islands (La Palma and El Hierro, Table 1), as a result of large erosion episodes (Mitchell et al., 2003). This might, in turn, affect the availability of suitable nearshore habitats for epi‐benthic sharks (Meyers et al., 2017). Abyssal barriers between adjacent islands, except between Lanzarote and Fuerteventura that share the same shelf, may also constrain connectivity between islands for fauna of limited pelagic dispersal (Brito et al., 2002).

**TABLE 1:** Geological and geomorphological characteristics of each of the seven main islands of the Canarian archipelago

	Island	Age (million years)	Coastal perimeter (km)	Coastal hard bottoms (%)	Distance to Africa (km)	Mean shelf width (km)	Total shelf area (Km^2^)	Hard bottoms (%)	Soft bottoms (%)
Eastern I.	Lanzarote	15.5	233.2	78.41	125	1.65^a^	1119.8^a^	43	57
	Fuerteventura	20.5	357.3	76.46	95			45	55
Central I.	Gran Canaria	14.5	289.6	76.02	196	1.42	412.1	16	84
	Tenerife	7.5	416.5	84.15	284	0.67	280.1	41	59
Western I.	La Gomera	12	117.1	86.25	333	0.71	83.9	24	76
	La Palma	1.5	187.2	90.84	416	0.46	87.3	37	63
	El Hierro	0.8	133.4	94.69	383	0.23	31.9	72	28

Note

Compilation based on information provided by Fernández‐Palacios and Martín‐Esquivel (2002) and our own data collection. Both the mean insular shelf width and the total shelf area are calculated from the 0 (sea level) down to the 50‐m‐depth isobath. Also, the percentages of hard and soft bottoms were calculated between the 0 and the 50‐m‐depth isobath, using available cartographies (www.miteco.gob.es/es/costas/temas/proteccion‐costa/ecocartografias/default.aspx), funded by the coastal national authority as the base for marine spatial planning.

^a^ Lanzarote and Fuerteventura share the same insular shelf.

In this study, we took advantage of the particular geological and geomorphological configuration of the Canary Islands to assess whether patterns in the occurrence and abundance of batoids varied between groups of islands arranged in an east‐to‐west gradient (i.e., eastern, central, and western islands), for data collected from shallow (<40 m) and deep waters (60–700 m). To shed light on these patterns, we also looked at differences in the abundances of shallow‐water batoids through a range of nearshore habitats, among groups of islands, and considered variation in a range of geomorphological attributes (e.g., shelf extension and availability of habitats). Finally, we sought to discuss the conservation implications of our results.

## 2 MATERIALS AND METHODS

### 2.1 Study region

The seven main islands of the Canarian archipelago were arranged into three groups, following an east‐to‐west gradient of varying proximity to the African coast, which, to some extent, corresponds to similarities in their geological histories and relevant geomorphological features, following a mantle plume “hotspot” volcanic origin (Table 1). Lanzarote and Fuerteventura, including the islet of *Lobos* and the *Chinijo* Archipelago (a group of four islets) north of Lanzarote, are the older islands, and share an extensive shelf. Subsequently, these islands (and islets) were categorized as the “eastern islands.” The “central islands” include Gran Canaria and Tenerife, old to middle‐age islands with moderately large and independent, insular shelfs. Finally, the islands of La Gomera, La Palma, and El Hierro, that is, the “western islands,” are the youngest islands of the archipelago, particularly El Hierro and La Palma, which are characterized by reduced and abrupted insular shelfs (Table 1).

### 2.2 Shallow water batoids

We compiled a database of Underwater Visual Counts (UVCs, Appendix S2) carried out across the entire Canary Islands during the last four decades. We firstly searched for every published study, by means of UVCs, targeting the entire assemblage of shallow water fishes (<40 m depth), using the Web of Science database. Several keywords were used, combined in various ways: “fish*,” “Canary*,” “visual,” and “count.” In addition, we included a technical report that contained a large quantity of UVCs at Fuerteventura Island (López‐Jurado et al., 1996). Most studies collected fish information via 100 m^2^ strip transects (8 of the 12 publications), although a number of studies collected data through 100 m^2^ stationary points (4 of the 12 publications; visual censuses are performed in a circular area with a radius of 5.6 m, Appendix S3). For every study, we extracted data on the abundance of batoids for each replicate, by taking advantage of data published in tables, figures, and appendices. UVCs carried out in the water column were ignored, that is, only UVCs directly performed on the seabed were considered. Studies limited to checklists, or exclusively providing qualitative abundances, were also discarded. We also annotated the type of habitat where UVCs took place, by considering four habitat types: reef, seagrass meadows, sandy bottoms, and sea‐cage fish farms (which are always above sandy bottoms). In addition, we downloaded 236 transects from the Reef Life Survey (RLS) portal (https://reeflifesurvey.com), a public, open‐access database, where data are collected through a citizen science program under strict scientific supervision (Edgar et al., 2020). Briefly, RLS divers perform 500 m^2^ transects; these data were standardized to 100 m^2^ by diving abundances by 5. Similarly, the dataset from Bosch et al. (2017), which used 40 m^2^ transects, was standardized to 100 m^2^ by multiplying abundances by 2.5. Overall, we compiled information from a total of 238 sites (2367 UVCs) across the entire archipelago. A larger effort, in terms of the total number of sites and UVCs, was carried out in the central islands (113 sites and 1426 counts) relative to both the eastern (66 sites and 409 counts) and western islands (59 sites and 532 counts). More research effort concentrated on reefs across the three island groups (Appendix S3).

Data were also compiled from a local citizen (community) science database (“Red Promar,” Government of the Canary Islands, www.redpromar.com). Citizens upload data on sightings of marine species (including the island, location, and date), which are then checked by local experts in marine biology. However, no information on the habitat of sightings is provided. Data can be freely downloaded. Overall, we downloaded 362 reports of batoids across the entire archipelago, mostly reported by recreational SCUBA divers, throughout the entire 2020 to the 1^st^ of June 2021 period. The two records of devil rays were grouped as *Mobula* spp., while we ignored species with just one record.

### 2.3 Deep water batoids

Data were collected during a field expedition led by the NGO Oceana, using a 21‐m‐long vessel (*Oceana Ranger*), between the 24th of August and the 8th of October 2009. Batoids inhabiting deep (60–700 m) waters were identified through deployments of a Remote Operated Vehicle (ROV, Seaeye Falcon DR), which incorporated a HD color video camera (480 TVL, minimum scene illumination 0.2 Lux). The horizontal field view was ~91° (Tilt ± 90°), and typically ranged between 1.5 and 1.7 m. Data were transmitted onboard through an optical fiber cable (14 mm). The ROV was towed by the vessel at a constant speed (~0.2 knots), following predesigned transects of varying dimensions according to the topographical peculiarities of each sampling site. A total of 41 ROV deployments, mostly between 2 and 5 h, were carried out at each of the seven main islands of the archipelago, as well as around the small islets north of Lanzarote (more information on methodology and selection of sites at https://eu.oceana.org/sites/default/files/euo/OCEANA_Propuestas_AMIE_Canarias_ESP.pdf). All sites were within Zones of Special Protection (EU Nature 2000 network, www.gobiernodecanarias.org/medioambiente/temas/biodiversidad/espacios_protegidos/red‐natura‐2000/red_natura_2000_en_canarias/). Videos were subsequently analyzed and batoids were identified and counted. If the same batoid was visualized on successive occasions, for example, a few seconds apart, the animal was only counted once. Initially, the effort was not balanced among the three groups of islands (13, 17, and 11 ROV deployments, for the western, central, and eastern islands, respectively). However, when the number of hours was accounted, and summed across each island group, the effort was much balanced with 39, 41, and 38 h of video recordings, respectively, for the western, central, and eastern islands.

### 2.4 Statistical analyses

To allow comparisons in taxonomic diversity across islands groups for the UVC data because of varying research effort among island groups, rarefraction curves for each island group were obtained, using the EstimateS package (Colwell, 2019). Both sample‐based and individual‐based rarefraction curves were obtained to represent how the number of species varied as a function of the number of counts and individuals, respectively, for each islands group (i.e., species density and species richness, respectively, Gotelli & Colwell, 2001); confidence intervals (95%) were calculated with a bootstrap procedure.

Differences in total batoid abundances between groups of islands were analyzed via generalized linear models (GLMs), implemented in the R statistical environment, via the MASS package (Venables & Ripley, 2002), separately for shallow (i.e., UVCs data) and deep (i.e., ROV deployments) waters. As response variables, we then considered the abundance of batoids per UVC and per ROV deployment, respectively. Because many batoid observations came from UVCs around fish farms, in the case of shallow waters, we carried out two separate GLMs, for all data and without data collected around fish farms. In the case of the UVC data, we complemented this initial analysis with a multivariate model that, in addition to island groups, included habitat types and the island shelf area, as a way of considering mechanisms operating at small (i.e., the habitat of each count) and large scale (i.e., at the insular scale). In all analyses, data were fitted through a “negative binomial” family error structure, and a “log” link function, which are ideal for overdispersed count data (White & Bennetts, 1996). We used the R “relevel” function to reorder levels, as a way of contrasting levels of island groups on responses. The assumptions of linearity and homogeneity of variances were checked through visual inspection of residuals and Q‐Q plots (Harrison et al., 2018). Chi‐squared (χ^2^) statistics tested whether frequencies in the observation of batoids, via UVCs, differed among habitats, and whether frequencies in reported batoids from the local public community science database “Red Promar” varied among the three island groups.

## 3 RESULTS

Eleven species of batoids were registered by means of three complementary data sources. According to the IUCN Red List of Threatened Species, three of these species are categorized as “Critically Endangered”: *Aetomylaeus bovinus* (Geoffroy St. Hilaire 1817), *Dipturus batis* (Linnaeus 1758), and *Myliobatis aquila* (Linnaeus 1758); three are “Endangered”: *Gymnura altavela* (Linnaeus 1758), *Mobula mobular* (Bonnaterre 1788), and *Rostroraja alba* (Lacepède 1803); two are “Vulnerable”: *Dasyatis pastinaca* (Linnaeus 1758) and *Raja maderenseis* (Lowe 1838); and two are “Data Deficient”: *Taeniurops grabata* (Geoffroy St. Hilaire 1809) and *Torpedo marmorata* (Risso 1810). Also, we observed a “Least Concern” species: *Bathytoshia lata* (Mitchill, 1815).

**FIGURE 1:**
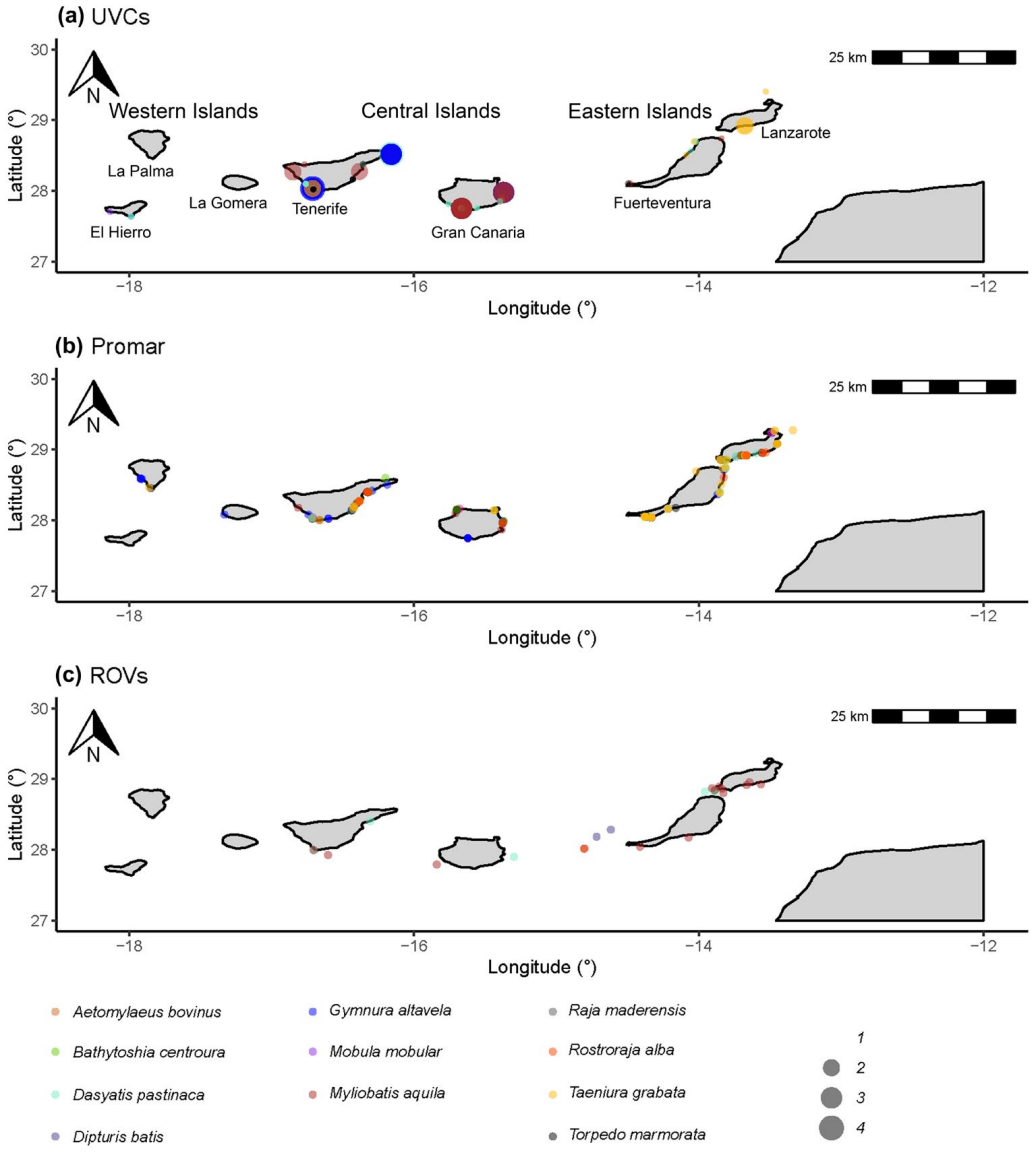
Maps detailing the occurrence (total number of observations) of batoids across the Canary Islands for data collected through (a) UVCs (*N* = 8 species), (b) a local community science database (“Red Promar,” *N* = 9 species), and (c) ROV deployments (*N* = 5 species)

**FIGURE 2:**
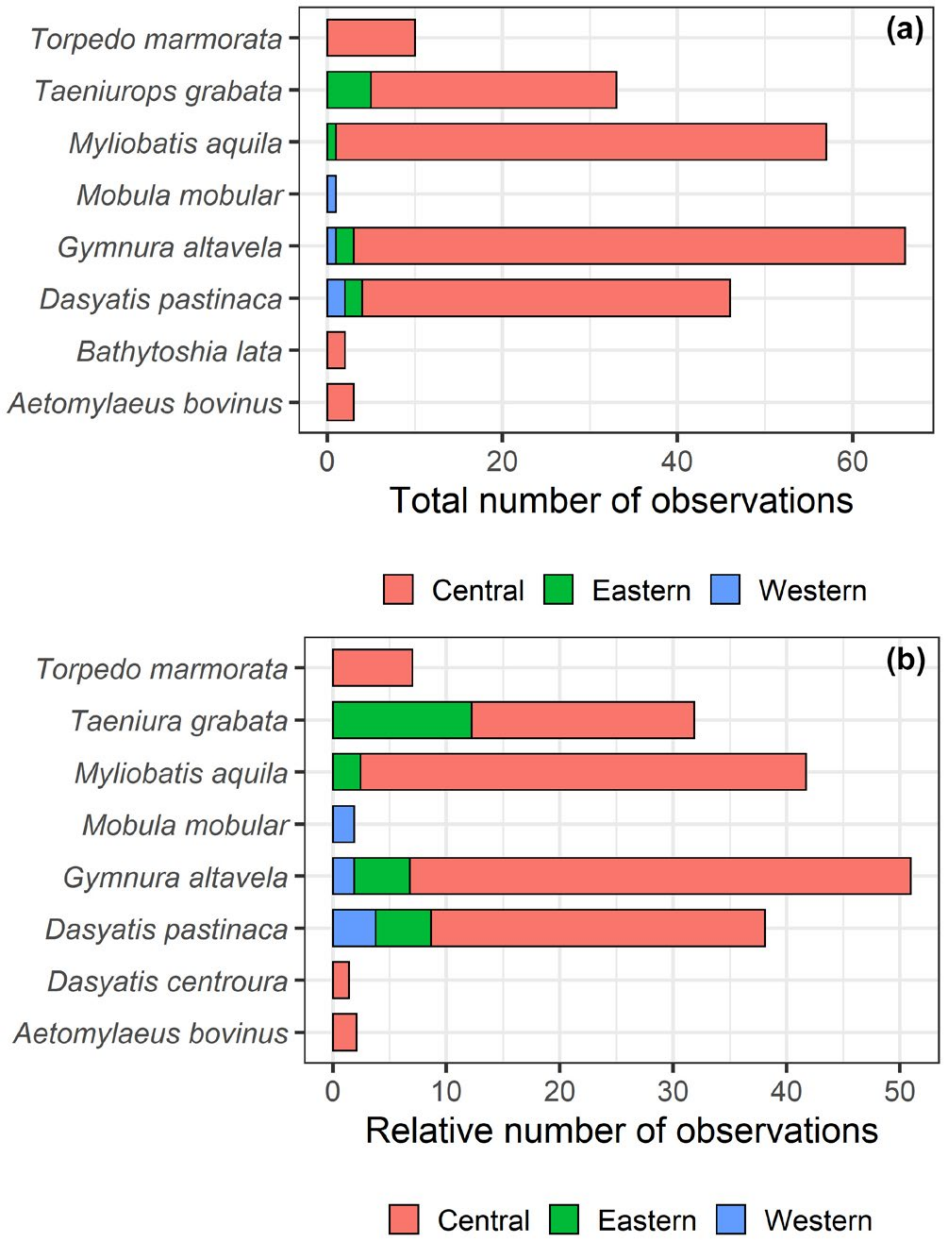
(a) Total and (b) relative (according to the number of fish counts, i.e., dividing by the number of counts and multiplying the result by 1000) number of observations of batoid species at the western, central, and eastern islands of the Canarian archipelago, identified through UVCs

**FIGURE 3:**
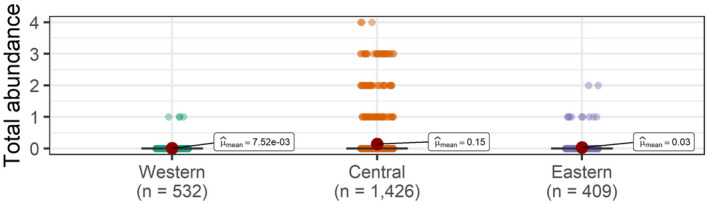
Total abundances of batoids at the western, central, and eastern islands of the Canarian archipelago. Each point represents the total abundance per replicate (UVC)

**FIGURE 4:**
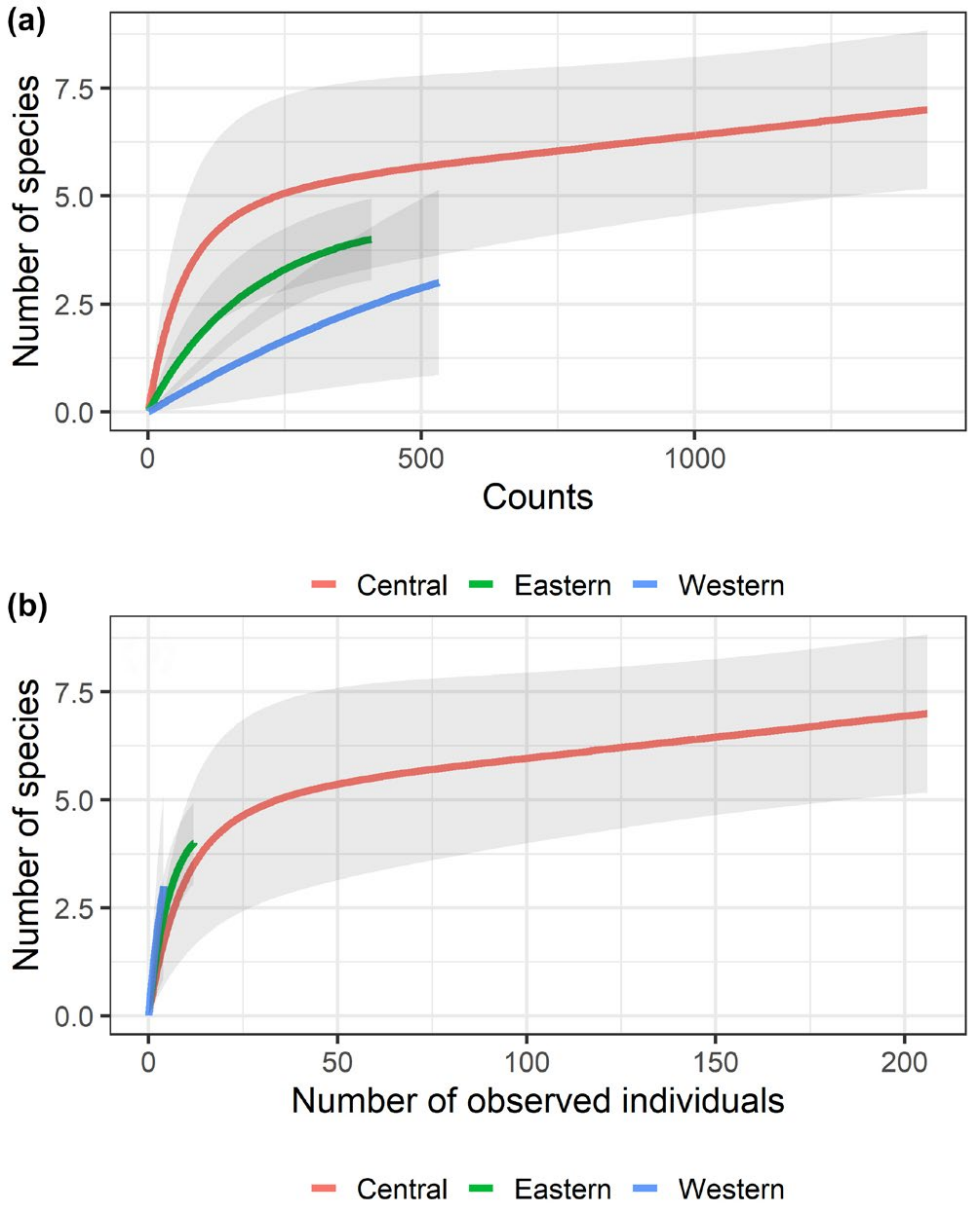
Rarefraction curves denoting increases in (a) species density with increasing sampling effort (UVCs) and (b) species richness with the number of observed individuals at the western, central, and eastern islands of the Canarian archipelago. Confidence intervals (95%) are grey‐shaded areas

**FIGURE 5:**
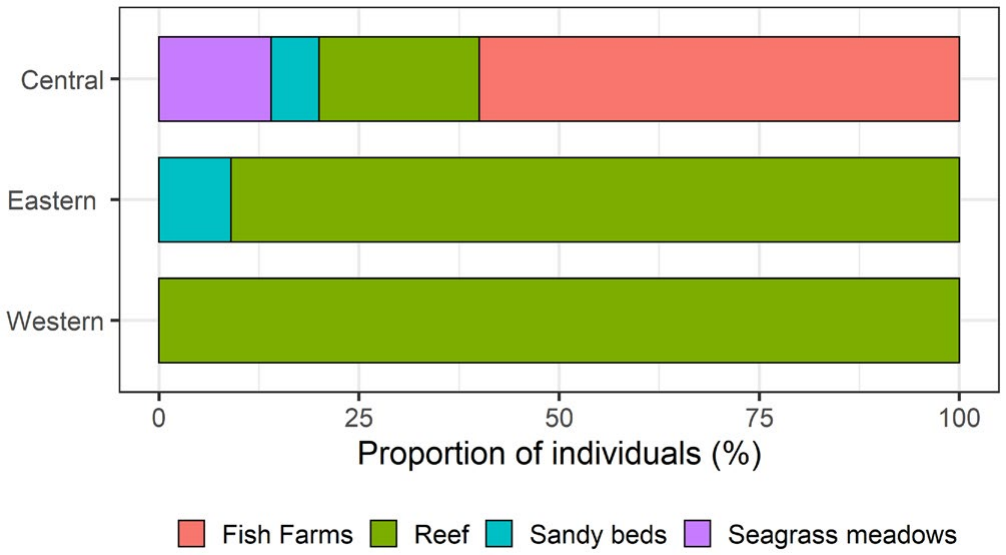
Proportion of batoids observed at each of the four habitat types at the western, central, and eastern islands of the Canarian archipelago via UVCs

A total of 226 records of batoids were collected via UVCs from shallow waters, belonging to a total of eight species (Figures 1a and 2). Overall, greater abundances (~one order of magnitude) were observed in the central and eastern islands, relative to the western islands (Figure 3, Table 2). A total of 4, 7, and 3 species were observed in the eastern, central, and western islands, respectively. For a similar amount of effort, the central islands showed a larger species density (Figure 4a). However, detection of species with increases in the number of observed individuals was similar among island groups (i.e., species richness, Figure 4b). When data from fish farms were not considered, we still observed greater abundances in the central and eastern than in the western islands (Table 2); however, abundances in the central islands did not differ relative to the eastern islands (Table 2). In the central and eastern islands, batoids were observed in a larger variety of habitats (Figure 5), relative to the western islands, where batoids were exclusively observed on reefs (Figure 5) because all counts were carried out on reefs (Appendix S3). Overall, ~52% of reported batoids were observed under sea‐cage fish farms, ~28% on reefs, ~12% on seagrass meadows, and ~7% on sandy bottoms, which resulted in statistically significant differences (χ^2^ = 82.2, *p* < 2.2e^−16^). Contrary to these patterns, the only individual of the charismatic giant devil ray, *Mobula mobular*, was observed in the western islands (Figure 2). These patterns were corroborated by the multivariate model, which not only identified larger abundances in the central islands and fish farms but also a significant, positive, increase in batoid abundances with the island shelf area (Table 3).

**TABLE 2:** Results of the GLM testing for differences in the abundance of batoids among the three groups of islands for data collected through UVCs (from all habitats and excluding data from fish farms)

Coefficients	Estimate	*Z*	*p*
Intercept	−3.44	−10.37	**<2e^−16^**
Central	1.52	4.33	**1.46e** ^−^ **^05^**
Western	−1.44	−2.32	**0.02**
Intercept	−1.92	−16.07	**<2e**−**^16^**
Eastern	−1.52	−4.33	**1.46e** ^−^ **^05^**
Western	−2.97	−5.51	**3.43e** ^−^ **^08^**
*UVCs (without data from fish farms)*			
Intercept	−3.42	−11.60	**<2e^−16^**
Central	0.09	0.27	.78
Western	−1.46	−2.48	.**01**
Intercept	−3.33	−21.35	**<2e** ^−^ **^16^**
Eastern	−0.09	−0.27	.78
Western	−1.55	−2.92	.**003**

Note

Significant *p*‐values (*p* < .05) are highlighted in bold. Reference levels are “Eastern” and “Central” islands, respectively, for each pair of comparisons.

**TABLE 3:** Results of the GLM testing for differences in the abundance of batoids among the three groups of islands, habitat types, and island shelf area, for data collected through UVCs

Coefficients	Estimate	*Z*	*p*
Intercept	−3.95	−9.95	**<2 e** ^−^ **^16^**
Eastern	−2.11	−4.25	**2.07 e** ^−^ **^5^**
Western	−1.09	−2.00	.**04**
Farm	4.14	10.98	**<2 e** ^−^ **^16^**
Reef	0.09	0.23	.81
Sand	0.99	2.24	.**02**
Shelf area	0.001	3.46	**5.37 e** ^−^ **^4^**

Note

Significant *p*‐values (*p* < .05) are highlighted in bold. Reference levels are “Eastern” and “Central” islands, respectively, for each pair of comparisons.

**FIGURE 6:**
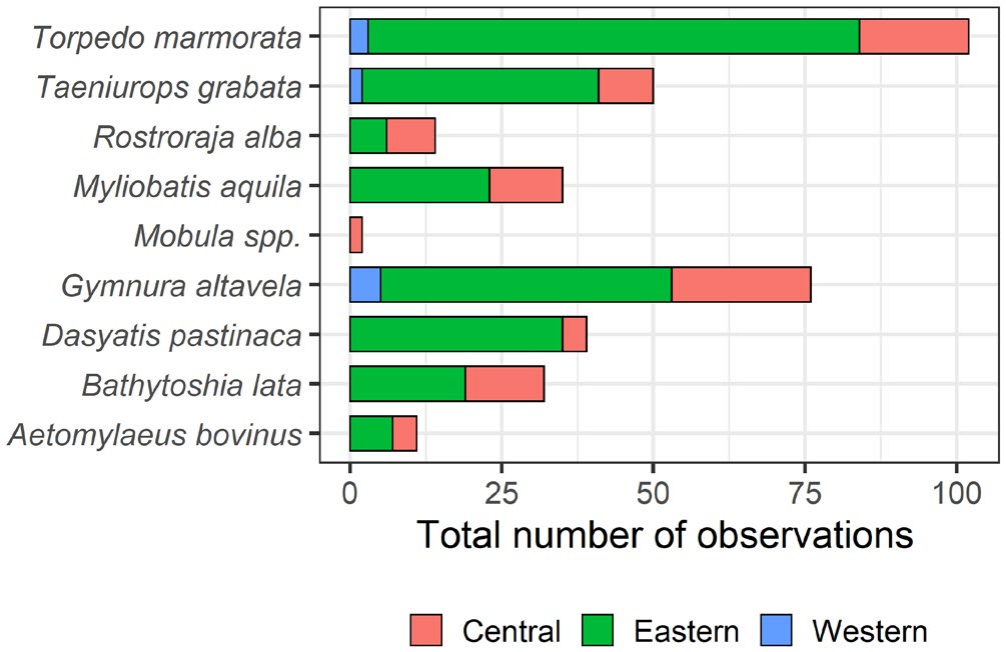
Total number of batoid observations at the western, central, and eastern islands of the Canarian archipelago, provided by the Red Promar citizen science database

From the 362 records of batoids provided from the local community science database (Figure 1b), a total of nine species were identified, with eight, nine, and three species from the eastern, central, and western islands, respectively. Observations were more frequently reported for both the eastern and central islands, relative to the western islands (Figure 6). Overall, ~71% of reported batoids were from the eastern islands, ~26% from the central islands, and only ~3% from the western islands, which resulted in statistically significant differences (χ^2^ = 397.3, *p* < 2.2e^−16^).

**FIGURE 7:**
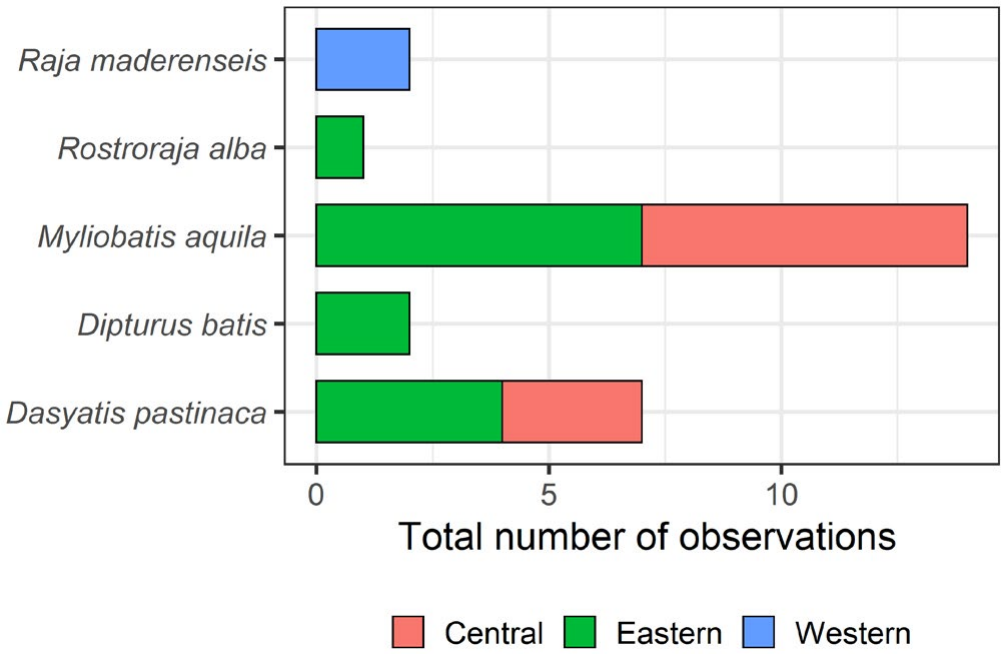
Total number of observations of batoid species inhabiting at the western, central, and eastern islands of the Canarian archipelago, identified through ROV deployments

**FIGURE 8:**
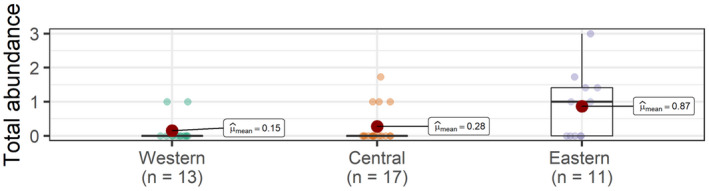
Total abundances of batoids at the western, central, and eastern islands of the Canarian archipelago. Each point represents a ROV deployment

A total of 26 batoids were observed at deep waters, belonging to a total of five species (Figures 1c and 7), with four, two, and one species from the eastern, central, and western islands, respectively. A total of 14 observations were between 400 and 500 m depth, 9 from <100 m depth, and 3 between 100 and 400 m depth. Significantly larger abundances of batoids were observed in the eastern islands, relative to the central and western islands (Figure 8, Table 4).

**TABLE 4:** Results of the GLM testing for differences in the abundance of batoids among the three groups of islands for data collected through the ROV

Coefficients	Estimate	*Z*	*p*
Intercept	−1.04	−2.02	.**04**
Eastern	1.53	2.23	.**02**
Western	−0.83	−0.88	.37
Intercept	−1.87	−2.36	.**01**
Central	0.83	0.88	.37
Eastern	2.36	2.59	.**009**

Note

Significant *p*‐values (*p* < .05) are highlighted in bold. Reference levels are “Central” and “Western” islands, respectively, for each pair of comparisons.

## 4 DISCUSSION

Patterns of occurrence and abundance of batoids across ocean basins are often attributed to large‐scale (>100 km) variation in environmental factors (e.g., through the Mediterranean, Follesa et al., 2019), while small‐scale variation in habitat features may account to explain local patterns of abundance (e.g., within estuaries; Possatto et al., 2016). This study demonstrated the existence of differences in the occurrence, abundance, and species density of batoids across the Canary Islands along an east to west gradient, a pattern that seems to relate to differences in the extension and configuration of shelfs of the different islands because of their varying geological histories. Importantly, this pattern was consistent for both shallow and deep waters via three complementary data sources, highlighting the benefits of complementary, open‐access data sources for the management and conservation of species of conservation concern (Edgar et al., 2020). Although the varying geological history and geomorphological differences of the islands are a plausible explanation for these patterns, the influence of large‐scale oceanographic variation across the archipelago (Davenport et al., 2002) as a covarying driver cannot be ruled out (Mazzei et al., 2021). In addition, an unbalanced research effort among islands may have also influenced patterns that here we outline, as we further discuss.

Insular shelfs on the eastern and central islands, that is, the mid‐ to older islands, are wider and more extensive than in the western islands (Table 1), potentially providing a more ample range of favorable habitats. A similar explanation was considered to describe sites of large abundances of the Thornback ray, *Raja clavata*, across the Mediterranean (Follesa et al., 2019). The western islands have a rockier coast (Table 1, mean % of coastal rocky bottoms of 77.43% ± 1.37, 80.08% ± 5.74, and 90.59% ± 4.22, for the eastern, central, and western islands, respectively), whereas low relief, protected, shallow water areas are sparse, which might be ideal places for batoid recruitment. Even though the overall extension of soft bottoms does not appear larger in the eastern and central islands compared to the western islands (Table 1), certain soft‐bottom habitats are less conspicuous in the western islands. In this sense, seagrass meadows are more developed in the eastern and central islands than in the western islands, where meadows constituted by the seagrass *Cymodocea nodosa* are very sparse and reduced (Barberá et al., 2005; Fabbri et al., 2015; Pavón‐Salas et al., 2000; de la Rosa et al., 2015). Most batoids typically inhabit soft bottoms, taking advantage of a compressed body that is ideal for this type of seabeds, for example, in terms of hiding (Farré et al., 2015). In the study region, small‐sized batoids have been previously observed in seagrass meadows, but at low densities (Espino et al., 2011). Hence, seagrass meadows do not appear to be a critical habitat for batoids. In brief, the wider continental shelfs of the eastern and central, relative to the western, islands provide more available area. Alternative explanations are plausible to clarify the lower occurrence and abundance of batoids in the western islands. A parallel mechanism, in this sense, may be the proximity to the western African coast. It is plausible that past and present colonization events by benthic batoids across the Canarian archipelago depend on distance to the nearby continental masses, following the classic “Island of Biogeography Theory” (MacArthur & Wilson, 2001), which predicts that increasing isolation result in lowered species richness (Hachich et al., 2020). In brief, the older and less isolated islands (eastern and central islands) have had more time to experience immigration/colonization events of marine biota of low dispersal capabilities (Hachich et al., 2020). In turn, this has been considered to explain the lower occurrences of angelshark (*S. squatina*) toward the westernmost islands (Meyers et al., 2017). In the study region, this seems to be particularly relevant, as abyssal barriers between adjacent islands (>2000 m depth), except between Lanzarote and Fuerteventura, may considerably constrain connectivity between islands for batoids with limited pelagic dispersal (di Santo & Kenaley, 2016; Elston et al., 2021). Batoids are species with direct development of embryos inside the mother (Dulvy & Reynolds, 1997; McEachran & Capapé, 1984). These species, moreover, lack “rafting” capacities (drifting in the water column associated with objects) as juveniles (sensu Hachich et al., 2020), a mechanism that facilitate short‐term dispersion among distant areas. In contrast, it is worth noting that the only three individuals of enigmatic devil rays, *Mobula* spp., were observed in the western and central islands. Devil rays are migratory fish with high dispersal capacities and tropical affinities (Couturier et al., 2012; Jaine et al., 2012) and, in turn, many sightings of this pelagic ray come from these islands, which have more tropical conditions compared to the eastern islands (Brito et al., 2002; Espino et al., 2018).

This study has corroborated that sea‐cage fish farms in the Canary Islands aggregate a large number of batoids of some species, when compared to other nearshore habitats (Tuya et al., 2005; Tuya, Sánchez‐Jerez, Dempster, et al., 2006) and other regions, for example, the Mediterranean (Dempster et al., 2005). Most sea‐cage fish farms (ca. 90%) are in the central islands (Gran Canaria and Tenerife) because of logistical reasons, with major exporting carriers in these two islands (www.gobiernodecanarias.org/pesca/temas/cultivos_marinos/). Initially, this could confound the macroecological pattern we describe in this study. However, when observations from fish farms were ignored, our results still identified differences in batoid abundances among groups of islands. More importantly, a potential bias to interpret our results may be an underestimation of the population abundances of batoids because of a low sampling effort on soft bottoms. Typically, most studies performing UVCs are implemented on rocky reefs, seagrass meadows, and artificial habitats (farms), that is, at habitats with a priori large abundances and diversity of coastal bonny fishes. In the Canary Islands, for example, certain species of batoids can be found on sandy bottoms, while being absent at adjacent rocky reefs (Tuya et al., 2019).

Another potential shortcoming of this study may be an unbalanced sampling effort among groups of islands. However, we believe this does not severely confound the main conclusion of this study. In this sense, the amount of effort for both UVCs and ROV deployments was even larger on the western (532 UVCs and 13 ROV deployments) than in the eastern islands (409 UVCs and 11 ROV deployments), which therefore rule this out as an explanation for the lower batoid abundances in the western islands. However, as indicated by the rarefraction curves, more sampling in the western and eastern islands is needed to capture a better picture of the batoid diversity there. It is true, however, that a larger number of fish counts (1426) came from the central islands (Gran Canaria and Tenerife), the most populated islands, which harbor local marine research centers and universities, and so a more direct access to the field to carry out any study by direct observation through SCUBA diving. With regard to data provided by the “Red Promar” citizen (community) science database, most observations come from recreational divers. Despite the western islands being less populated than the central and eastern islands, SCUBA diving is of great popularity at the westernmost island (El Hierro), with nine diving centers and >20,000 divers per year, which, to a certain extent, rules out the potential low observation effort at the western islands (Meyers et al., 2017). In terms of the temporal frame of the data we here analyzed, it is worth mentioning that no publication from the study region has demonstrated a range shift for any batoid species (Báez et al., 2019). Despite a temporal comparison would be ideal to assess temporality in the presence of batoids in the Canary Islands, most UVC data (ca. 80%) come from the last two decades, while the ROV data and citizen sighting data are from the last decade. In brief, all this limits such temporal analysis. Also, our data did not analyze any seasonal pattern, a factor that we overlooked and should be addressed in the future.

Traditionally, most data on the presence and abundance of batoids are derived from fisheries data, but most of these species are discarded and their catches are not reported, nor recorded in the artisanal fishery statistics, generating an important deficit of information about their distribution and status. In the last decades, fisheries‐independent data sources collected by scientists via UVCs, BRUVs, and ROVs (Caldwell et al., 2016; Espinoza et al., 2020; Moored et al., 2011) have been increasing. Data collected by community scientists provide a cost‐effective alternative, which can be used to understand species distributions, particularly for charismatic species of low abundances, such as certain elasmobranchs (Edgar et al., 2020; Giovos et al., 2019; Hussey et al., 2013; Jaine et al., 2012). When ecological data are sparse, difficult, and expensive to obtain, “Local Ecological Knowledge,” through citizen science programs, provides an alternative and complementary data source. In our case study, it is worth noting that community scientists identified a total of nine species, while UVCs only accounted for eight species.

The consistency in our results, for shallow and deep waters, seems to outweigh the limitations intrinsic to each sampling technique. For example, highly migratory batoids, such as devil rays, are difficult to spot while performing UVCs. Similarly, the limited field view of cameras in ROVs can underestimate the abundance of elasmobranchs, even though batoids are bottom‐dwelling species compared to highly mobile sharks (Follesa et al., 2019).

The main direct threat to batoids is fisheries exploitation, particularly via bottom trawling, leading in some cases to local extirpation (Sguotti et al., 2016; Ward & Myers, 2005). However, in the Canary Islands, bottom trawling has hardly been practiced, mainly due to the reduced insular shelfs. This may be a key explanation for the large diversity and abundance of rays and sharks in the archipelago (Brito et al., 2002). Moreover, there are no targeted fisheries for batoids, but incidental catches may be a concern in the small‐scale fishing fleet, especially when “Cazonal,” or trammel nets, and bottom longlines are used (Franquet & Brito, 1995; Mendoza et al., 2018). The local fishery is typically artisanal and essentially composed of small (<15 m) vessels using hooks and lines and traps, which mainly target ray‐finned fishes (Tuya et al., 2006). The capture of elasmobranchs, in particular by spearfishermen, is negligible in the study region (Jiménez‐Alvarado et al., 2020). Hence, our results are not confounded by varying levels of fishing effort among groups of islands. It should be noted that, in this study, less than half of the batoids species known to occur in the Canary Islands (Báez et al., 2019; Brito et al., 2002; Appendix S1) were recorded, even though our data covered a broad spectrum of sites, depths, and habitats. Estimating abundance patterns are crucial for any effective conservation initiative for batoids, including the establishment of protected areas for conservation at different geographic (e.g., insular) scales. Collecting data on elasmobranch species are especially difficult because most are sparse, mobile, and display ontogenetic shifts. Although there are limited resources and funding to undertake such sampling, this study has demonstrated that a combination of data sources can shed light on elasmobranch occurrence and abundance patterns across regional scales.

In the Canary Islands, some batoid species are coastal and use shallow waters, making them more susceptible to a combination of fishing pressure, habitat degradation, and urban development. Several batoids are known to display site fidelity and philopatry, that is, individuals frequently return to, or stay in their home ranges, birthplaces, or other specific localities (Chapman et al., 2015), which can structure their populations over fine geographical scales (Flowers et al., 2016). Given the low dispersal (locomotory) capacity of most benthic batoids (Di Santo & Kenaley, 2016; Elston et al., 2021), despite some exceptions (Boggio‐Pasqua et al., 2019), our data suggest that, rather than adopting an archipelago‐wide strategy as a single unit, actions and targets specific to each island should be developed, as part of any recovery or management plans. This outcome reinforces the idea of taxon specificities, that is, taxon dependencies, when establishing conservation actions. In brief, each group of islands should have its tailored strategy under a common overall aim to maintain the populations in an optimal conservation status. Moreover, species’ habitat, ecology, distribution, behavior, and evolution for all size classes need to be better understood and taken into consideration to complement conservation and management strategies. More specifically, further research efforts should be focused on genetic and movement studies to provide insights into the current and past levels of connectivity of batoids in the Canary Islands. Because ray abundances and habitat use can be explained by predator abundance, for example, sharks (Bond et al., 2019; Sherman et al., 2020), this effect should be also considered, particularly since abundance and movements of sharks on shallow waters of the study region are ignored. This will be key to move forward into efficient conservation of batoids in this region.

## CONFLICT OF INTEREST

None declared.

## AUTHOR CONTRIBUTIONS

**Fernando Tuya:** Conceptualization (lead); Investigation (lead). **Ricardo Aguilar:** Investigation (equal); Resources (equal); Visualization (equal). **Fernando Espino:** Investigation (supporting); Supervision (equal); Visualization (equal). **Nestor E. Bosch:** Conceptualization (equal); Data curation (equal); Formal analysis (equal); Writing‐original draft (equal). **Eva K. M. Meyers:** Conceptualization (equal); Investigation (equal); Writing‐original draft (equal). **David Jiménez‐Alvarado:** Formal analysis (supporting); Investigation (supporting); Software (supporting); Validation (supporting); Writing‐review & editing (supporting). **Jose J. Castro:** Data curation (equal); Funding acquisition (equal); Writing‐review & editing (equal). **Francisco Otero‐Ferrer:** Data curation (equal); Formal analysis (equal); Visualization (equal). **Ricardo Haroun:** Conceptualization (equal); Funding acquisition (equal); Writing‐original draft (equal).

## Supporting information

Appendix S1‐S3

## Data Availability

All data and R script to perform analysis are stored at https://github.com/ftuya/Batoids‐of‐Canary‐Islands.
