# Supplementary material for: Differences in the occurrence and abundance of batoids across an oceanic archipelago using complementary data sources: Implications for conservation
Source: Ecol Evol. 2021 Nov 18;11(23):16704–15. doi: 10.1002/ece3.8290 (PMC8668743; doi:10.1002/ece3.8290)
Supplement: Supplementary file 1 — Appendix S1‐S3 [file ECE3-11-16704-s001.docx]

Appendix 1. Checklist and taxonomy of batoid species present in the Canary Islands.

| Order | Family | Species | Authority |
| --- | --- | --- | --- |
| Torpediniformes | Torpedinidae | *Torpedo marmorata* | Risso, 1810 |
|  |  | *Tetronarce nobiliana* | (Bonaparte, 1835) |
| Rhinopristiformes | Rhinobatidae | *Rhinobatos rhinobatos* | (Linnaeus, 1758) |
| Rajiformes | Arhynchobatidae | *Bathyraja richardsoni* | (Garrick, 1961) |
|  | Rajidae | *Dipturus batis* | (Linnaeus, 1758) |
|  |  | *Dipturus oxyrinchus* | (Linnaeus, 1758) |
|  |  | *Leucoraja circularis* | (Couch, 1838) |
|  |  | *Raja brachyura* | Lafont, 1871 |
|  |  | *Raja clavata* | Linnaeus, 1758 |
|  |  | *Raja montagui* | Fowler, 1910 |
|  |  | *Rajella barnardi* | (Norman, 1935) |
|  |  | *Rajella ravidula* | (Hulley, 1970) |
|  |  | *Rostroraja alba* | (Lacepède, 1803) |
| Myliobatiformes | Dasyatidae | *Bathytoshia lata* | (Garman, 1880) |
|  |  | *Dasyatis pastinaca* | (Linnaeus, 1758) |
|  |  | *Pteroplatytrygon violacea* | (Bonaparte, 1832) |
|  |  | *Taeniurops grabatus* | (Geoffroy Saint-Hilaire, 1817) |
|  | Gymnuridae | *Gymnura altavela* | (Linnaeus, 1758) |
|  | Myliobatidae | *Aetomylaeus bovinus* | (Geoffroy Saint-Hilaire, 1817) |
|  |  | *Myliobatis aquila* | (Linnaeus, 1758) |
|  | Mobulidae | *Mobula alfredi* | (Krefft, 1868) |
|  |  | *Mobula birostris* | (Walbaum, 1792) |
|  |  | *Mobula mobular* | (Bonnaterre, 1788) |
|  |  | *Mobula tarapacana* | (Philippi, 1892) |

Appendix 2. Compiled studies targeting abundances of shallow-water fish faunas across the Canary Islands through UVCs (studies ordered chronologically). N denotes the number of replicated UVCs; the technique, i.e. either belt transects or stationary points, is included, as well as the area of UVCs.

| Title | Authors | Year | Publication |
| --- | --- | --- | --- |
| A visual assessment of the inshore fishes and fishery resources off El Hierro, Canary Islands: A baseline survey (N=369, 100 m^2^ point counts) | Bortone, S.A., van Tassell, J., Brito, A., Falcon, J.M., Bundrick, C.M. | 1991 | Scientia Marina  53: 529-541 |
| Structure of and relationships within and between the littoral, rock- substrate fish communities off four islands in the Canarian Archipelago (N=562, 100 m^2^ point counts) | Falcón, J. M., Bortone, S. A., Brito, A., & Bundrick, C. M. | 1996 | Marine Biology  125: 215-231 |
|  |  |  |  |
| Actuación de viabilidad para la estabilización de la población atlántica de foca monje (*Monachus monachus)*  (N= 62, 100 m^2^ point counts) | López-Jurado, L.F., Aparicio, F., Cedenilla, M.A., Espino, F., Fernández, J., Hernández, V., Herrera, R., Herrero, R., Hildebrandt, S., Moreno, T., Suárez, J., Quintana, S. | 1996 | Unpublished report |
| A visual survey of the inshore fish communities of Gran Canaria (Canary Islands) (N=210, 100 m^2^ point counts) | Hajagos, J., & van Tassel, J. | 2001 | Arquipélago  18A: 97-106 |
|  |  |  |  |
| Evaluación de las comunidades ícticas litorales de la zona propuesta como Reserva Marina Gando-Arinaga (Gran Canaria, Islas Canarias) mediante muestreos visuales (N=88, 100 m^2^ belt transects) | Tuya, F, Reuss, G.M., Martín, J. A., Luque, A. | 2004 | Ciencias Marinas  30: 259-278 |
| Relationships between rocky-reef fish assemblages, the sea urchin *Diadema antillarum* and macroalgae throughout the Canarian Archipelago (N=288, 100 m^2^ belt transects) | Tuya, F., Boyra, A., Sanchez-Jerez, P., Barbera, C., Haroun, R.J. | 2004 | Marine Ecology Progress Series  278: 157-169 |
|  |  |  |  |
| Vertical variability of wild fish assemblages around sea-cage fish farms: Implications for management (N=24, 100 m^2^ point counts) | Dempster, T., Fernandez-Jover, D., Sanchez-Jerez, P., Tuya, F., Bayle-Sempere, J., Boyra, A., Haroun, R.J. | 2005 | Marine Ecology Progress Series  304: 15-29 |
|  |  |  |  |
| Multivariate analysis of the bentho-demersal ichthyofauna along soft bottoms of the Eastern Atlantic: Comparison between unvegetated substrates, seagrass meadows and sandy bottoms beneath sea-cage fish farms (N=120, 100 m^2^ belt transects) | Tuya, F., Boyra, A., Sanchez-Jerez, P., Haroun, R.J. | 2005 | Marine Biology  147: 1229–1237 |
|  |  |  |  |
| Changes in demersal wild fish aggregations beneath a sea-cage fish farm after the cessation of farming (N=192, 100 m^2^ belt transects) | Tuya, F., Sanchez-Jerez, P., Dempster, T., Boyra, A., Haroun, R. | 2006 | Journal of Fish Biology  69: 682-697 |
|  |  |  |  |
| Ictiofauna asociada a las praderas de *Cymodocea nodosa* en las Islas Canarias (Atlántico centro oriental): Estructura de la comunidad y función de “guardería” (N=84, 100 m^2^ transect trawls) | Espino, F., Tuya, F., Brito, A., Haroun, R. J. | 2011 | Ciencias Marinas  37: 157–174 |
|  |  |  |  |
| Ecological structure and function differ between habitats dominated by seagrasses and green seaweeds (N=96, 100 m^2^ belt transects) | Tuya, F., Png-González, L., Riera, R., Haroun, R., Espino, F. | 2014 | Marine Environmental Research  [98](https://www.sciencedirect.com/science/journal/01411136/98/supp/C): 1-13 |
|  |  |  |  |
| “How” and “what” matters: Sampling method affects biodiversity estimates of reef fishes (N=12, 40 m^2^ belt transects)  Niche and neutral assembly mechanisms contribute to latitudinal diversity gradients in reef fishes (N=25, 100 m^2^ belt transects) | Bosch, N. E., Gonçalves, J. M.  S., Erzini, K., Tuya, F.  Bosch, N.E., Wernberg, T., Langlois, T.J., Smale, D.A., Moore, P.J., Franco, J.N., Thiriet, P., Feunteun, E., Riberiro, C., Neves, P., Freitas, R., Filbee-Dexter, K., Norderhaug, K.M., Garcıa, A., Otero-Ferrer, F., Espino, F., Haroun, R., Lazzari, N. Tuya, F. | 2017  2021 | Ecology and Evolution  7: 4891-4906  Journal of Biogeography  DOI10.1111/jbi.14237 |
|  |  |  |  |
|  |  |  |  |
|  |  |  |  |
|  |  |  |  |

Appendix 3. Number and proportion of UVCs on each nearshore habitat across islands groups. Number and proportion of UVCs according to point counts and belt transects across islands groups.

| Island Group | Habitat | Total number of counts | Proportion |
| --- | --- | --- | --- |
| Central | Seagrass meadow | 194 | 13% |
|  | Farms | 104 | 7% |
|  | Sandy bottoms | 208 | 14% |
|  | Rocky reefs | 920 | 66% |
| Eastern | Seagrass meadow | 74 | 18% |
|  | Farms | 8 | 2% |
|  | Sandy bottoms | 19 | 4% |
|  | Rocky reefs | 308 | 76% |
| Western | Seagrass meadow | 0 | 0 |
|  | Farms | 0 | 0 |
|  | Sandy bottoms | 0 | 0 |
|  | Rocky reefs | 532 | 100% |
| Central | Point counts | 696 | 49% |
|  | Belt transects | 730 | 51% |
| Eastern | Point counts | 162 | 39% |
|  | Belt transects | 247 | 61% |
| Western | Point counts | 369 | 69% |
|  | Belt transects | 163 | 31% |
